# Supplementary figures and images for: Longitudinal Profiling of the Macaque Vaginal Microbiome Reveals Similarities to Diverse Human Vaginal Communities
Source: mSystems. 2021 Apr 27;6(2):e01322-20. doi: 10.1128/mSystems.01322-20 (PMC8092128; doi:10.1128/mSystems.01322-20)

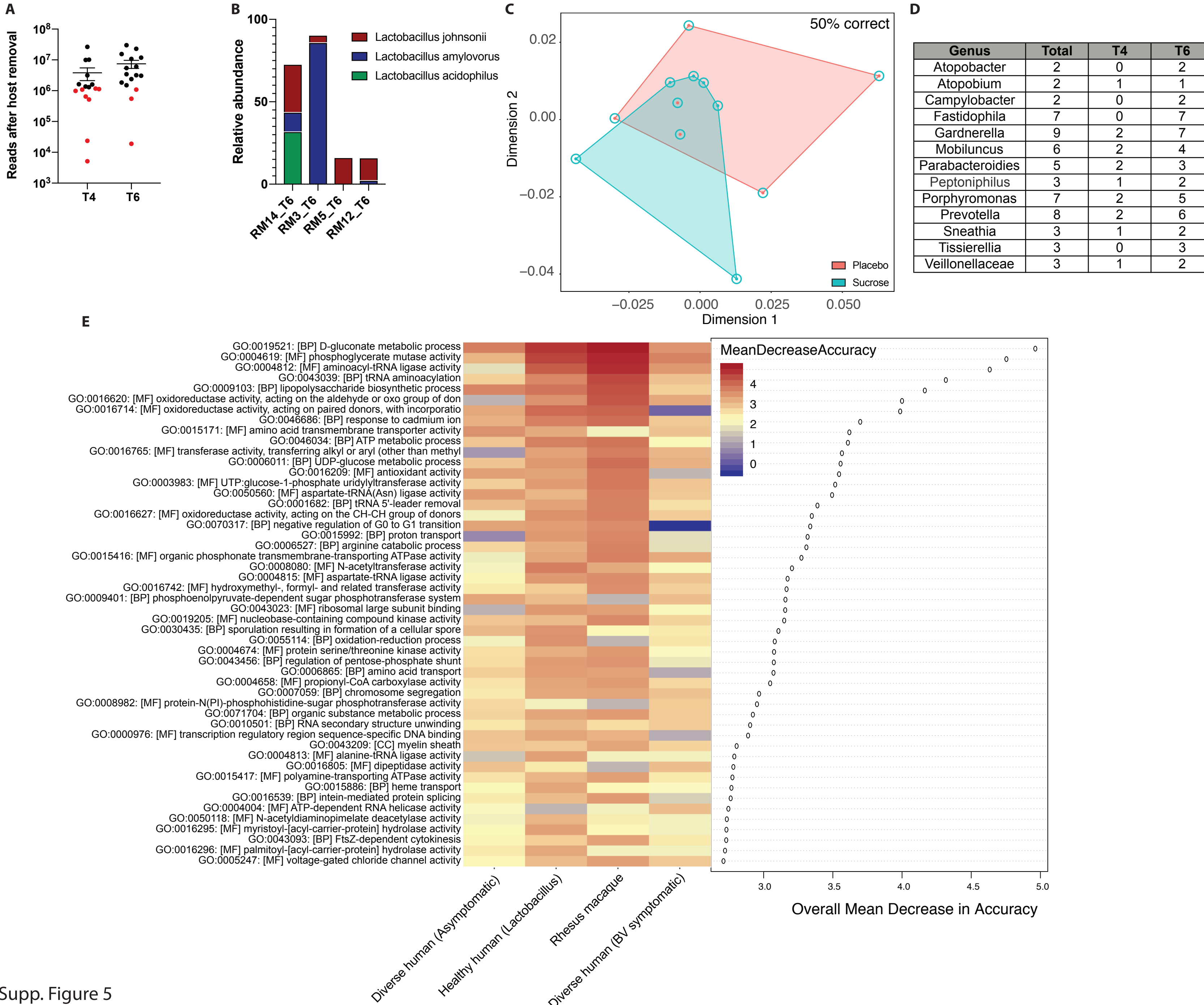

Supp. Figure 5

Supplement: FIG S5 [file mSystems.01322-20-sf005.pdf]
