# Supplementary material for: Longitudinal Profiling of the Macaque Vaginal Microbiome Reveals Similarities to Diverse Human Vaginal Communities
Source: mSystems. 2021 Apr 27;6(2):e01322-20. doi: 10.1128/mSystems.01322-20 (PMC8092128; doi:10.1128/mSystems.01322-20)
Supplement: TABLE S2 [file mSystems.01322-20-st002.pdf]

Bin Id	Marker lineage	Completeness	Contamination	Most similar genome	Distance	Common Kmer	Host	Source
RM4_T6_B4	o__Clostridiales (UID1120)	87.35	1.6	Anaerococcus vaginalis strain MGYG-HGUT-02315	0.186761	10	Human	Gut
RM13_T6_B6	o__Lactobacillales (UID355)	96.45	1.64	Atopobacter sp. AH10	0.02	501	Macaca fascicularis	viginal environment of primate
RM3_T6_B5	o__Lactobacillales (UID355)	96.82	1.64	Atopobacter sp. AH10	0.0204789	482	Macaca fascicularis	vaginal environment of primate
RM1_T6_B10	p__Actinobacteria (UID2112)	100	0	Atopobium deltae strain DNF00019	0.11	47	Human	vagina
RM12_T6_B7	p__Actinobacteria (UID2112)	96.77	0	Atopobium minutum strain DSM 20586	0.24	3	No listed	No listed
RM3_T6_B4	f__Porphyromonadaceae (UID2622)	95.77	0.31	Bacteroidales bacterium KA00251	0.186761	10	Human	No listed
RM10_T6_B4	o__Clostridiales (UID1120)	96.74	0.7	Bacteroides] coagulans strain DSM 20705	0.16	16	No listed	No listed
RM1_T6_B19	k__Bacteria (UID2372)	93.49	0	Bacteroidetes bacterium strain B12_G6	0.263022	2	deep-sea hydrothermal vent	sediments from dive
RM4_T4_B10	f__Porphyromonadaceae (UID2622)	94.58	0	Barnesiella sp. An55 strain An55	0.23011	4	Chicken, Gallus gallus	caecum
RM10_T6_B6	c__Epsilonproteobacteria (UID3065)	98.28	0.74	Campylobacter sputorum biovar sputorum strain NCTC12475	0.15	22	No listed	No listed
RM14_T6_B2	c__Epsilonproteobacteria (UID3065)	98.77	0.74	Campylobacter sputorum subsp. bubulus strain NCTC12474	0.16	19	No listed	No listed
RM1_T6_B11	o__Clostridiales (UID1212)	83.5	0	Clostridiales bacterium KA00134	0.26	2	Human	vagina
RM10_T6_B7	o__Clostridiales (UID1120)	88.77	0	Clostridiales bacterium KA00274	0.22	5	Human	Vagina
RM13_T6_B10	o__Clostridiales (UID1120)	88.06	0	Clostridiales bacterium KA00274	0.22	5	Human	Vagina
RM13_T6_B12	o__Clostridiales (UID1120)	84.74	0	Clostridiales bacterium KA00274	0.24	3	Human	Vagina
RM14_T6_B4	o__Clostridiales (UID1120)	81.21	0.71	Clostridiales bacterium KA00274	0.21	6	Human	Vagina
RM15_T6_B3	o__Clostridiales (UID1120)	88.77	0	Clostridiales bacterium KA00274	0.22	5	Human	Vagina
RM3_T6_B7	o__Clostridiales (UID1120)	88.59	0.71	Clostridiales bacterium KA00274	0.210897	6	Human	Vagina
RM10_T6_B9	k__Bacteria (UID2329)	100	0	Enterococcus faecium strain VRECG18	0.19	9	Human	Stool
RM7_T4_B9	o__Clostridiales (UID1212)	83.45	0	Eubacteriaceae bacterium CM5	0.23011	4	No listed	No listed
RM12_T6_B3	o__Clostridiales (UID1212)	87.47	0.67	Finegoldia magna strain 12T273	0.145	24	Human	Othopedic joint
RM11_T6_B3	f__Bifidobacteriaceae (UID1458)	96.61	0	Gardnerella sp. KA00735	0.2	7	Human	Vagina
RM12_T6_B6	f__Bifidobacteriaceae (UID1458)	96.61	0	Gardnerella sp. KA00735	0.2	7	Human	Vagina
RM4_T6_B6	f__Bifidobacteriaceae (UID1458)	96.84	0	Gardnerella sp. KA00735	0.191731	9	Human	Vagina
RM3_T6_B9	f__Bifidobacteriaceae (UID1458)	89.57	0.23	Gardnerella sp. KA00735	0.197292	8	Human	Vagina
RM5_T6_B1	f__Bifidobacteriaceae (UID1458)	96.61	0	Gardnerella sp. KA00735	0.203604	7	Human	Vagina
RM13_T4_B8	f__Bifidobacteriaceae (UID1458)	94.55	0	Gardnerella vaginalis 5-1	0.2	7	Human	Vagina
RM16_T6_B1	f__Bifidobacteriaceae (UID1458)	99.76	0.45	Gardnerella vaginalis 5-1	0.2	8	Human	Vagina
RM6_T6_B1	f__Bifidobacteriaceae (UID1458)	87.31	0.72	Gardnerella vaginalis strain PSS_7772B	0.210897	6	Human	Urine
RM4_T4_B9	f__Spirochaetaceae (UID2535)	97.12	1.05	Gardnerella vaginalis strain UGent 06.41	0.263022		Human	Vagina
RM1_T6_B8	f__Spirochaetaceae (UID2535)	97.12	1.05	Gardnerella vaginalis strain UGent 06.41	0.263022	2	Human	vagina
RM13_T6_B9	p__Firmicutes (UID1022)	95.95	0	Lactobacillus helveticus strain 459_LHEL	0.26	2	Human	No listed
RM2_T6_B2	f__Actinomycetaceae (UID1531)	80.2	1.42	Mobiluncus mulieris ATCC 35243	0.170926	14	Human	Urogenital tract, vagina
RM7_T4_B5	f__Actinomycetaceae (UID1531)	83.29	1.58	Mobiluncus mulieris FB024-16	0.108487	54	Human	Urogenital tract; Vaginal
RM4_T6_B2	f__Actinomycetaceae (UID1531)	99.05	1.54	Mobiluncus mulieris FB024-16	0.154223	20	Human	Urogenital tract; Vaginal
RM14_T6_B1	f__Actinomycetaceae (UID1531)	88.63	1.11	Mobiluncus mulieris strain NCTC11497	0.16	18	Human	Vagina
RM3_T6_B2	f__Actinomycetaceae (UID1531)	98.1	1.49	Mobiluncus mulieris strain NCTC11819	0.151946	21	No listed	No listed
RM8_T4_B1	f__Actinomycetaceae (UID1531)	80.58	1.26	Mobiluncus mulieris strain NCTC11819	0.0852783	91	Human	No listed
RM16_T6_B2	p__Actinobacteria (UID2112)	95.16	0	Paenibacillus stellifer DSM 14472	0.26	2	No listed	No listed
RM10_T6_B5	f__Porphyromonadaceae (UID2622)	94.89	0	Parabacteroides distasonis	0.24	3	No listed	No listed
RM13_T4_B4	f__Porphyromonadaceae (UID2622)	94.89	0	Parabacteroides distasonis	0.24	3	Human	Feces
RM13_T6_B8	f__Porphyromonadaceae (UID2622)	94.89	0	Parabacteroides distasonis	0.24	3	Human	Feces
RM1_T6_B9	f__Porphyromonadaceae (UID2622)	94.89	0	Parabacteroides distasonis	0.243761	3	Human	faeces
RM7_T4_B8	f__Porphyromonadaceae (UID2622)	94.89	0	Parabacteroides distasonis	0.243761	3	No listed	Faeces
RM1_T6_B13	o__Clostridiales (UID1120)	95.1	0	Peptoniphilus sp. KHD4	0.044	244	Human	vagina
RM13_T6_B5	o__Clostridiales (UID1212)	91.5	0	Peptoniphilus sp. oral taxon 836 str. F0141	0.2	7	Human	Oral
RM4_T4_B8	o__Clostridiales (UID1212)	91.5	0	Peptoniphilus sp. oral taxon 836 str. F0141	0.186761	10	Human	Oral
RM4_T6_B3	f__Porphyromonadaceae (UID2622)	99.61	0.16	Porphyromonas asaccharolytica PR426713P-I	0.154223	20	Human	Urogenital tract; Vaginal
RM10_T6_B3	f__Porphyromonadaceae (UID2622)	93.95	0.42	Porphyromonas bennonis DSM 23058	0.16	17	Human	Skin abcess
RM15_T6_B1	f__Porphyromonadaceae (UID2622)	95.05	0.31	Porphyromonas bennonis DSM 23058	0.16	17	Human	Skin abcess
RM13_T4_B1	p__Bacteroidetes (UID2605)	87.94	0.16	Porphyromonas somerae strain KA00683	0.26	2	Human	Vagina
RM13_T6_B3	p__Bacteroidetes (UID2605)	95.71	0	Porphyromonas somerae strain KA00683	0.26	2	Human	Vagina
RM7_T4_B3	g__Prevotella (UID2721)	98.57	0.12	Prevotella bivia DNF00188	0.23011	4	Human	Vagina
RM12_T6_B2	g__Prevotella (UID2721)	96.61	0.12	Prevotella buccalis strain UMB0536	0.23	4	Human	catheter
RM4_T6_B1	g__Prevotella (UID2721)	96.41	0.33	Prevotella buccalis strain UMB0536	0.0543589	190	Human	Catheter
RM10_T6_B1	g__Prevotella (UID2721)	93.1	0.12	Prevotella buccalis strain UMB0536	0.068	134	Human	Cathter
RM4_T4_B7	g__Prevotella (UID2721)	97.14	0	Prevotella pleuritidis F0068	0.219531	5	No listed	No listed
RM11_T6_B2	g__Prevotella (UID2721)	88.41	0	Prevotella timonensis strain DNF00076	0.26	2	Human	Vagina
RM1_T6_B6	g__Prevotella (UID2721)	96.61	0	Prevotella timonensis strain DNF00076	0.243761	3	Human	vagina
RM2_T6_B1	g__Prevotella (UID2721)	92.38	0.4	Prevotella timonensis strain DNF00076	0.263022	2	Human	faeces
RM15_T6_B4	k__Bacteria (UID2329)	100	0	Sneathia sp. Sn35	0.21	6	Human	Amniotic fluid
RM10_T4_B1	k__Bacteria (UID2329)	93.26	0	Sneathia sp. Sn35	0.197292	8	Human	Amniotic fluid
RM13_T4_B6	o__Clostridiales (UID1212)	82.33	0	Streptococcus sp. HMSC061D01	0.21	6	Human	throat
RM1_T6_B14	o__Clostridiales (UID1125)	84.6	0.61	Tissierellia bacterium KA00581	0.18	11	Human	vagina
RM13_T6_B11	o__Clostridiales (UID1125)	97.41	0.61	Tissierellia bacterium KA00581	0.15	20	Human	Vagina
RM4_T6_B8	o__Clostridiales (UID1125)	98.02	0.61	Tissierellia bacterium KA00581	0.164662	16	Human	Vagina
RM13_T4_B3	f__Spirochaetaceae (UID2535)	97.12	1.05	Treponema sp. strain W1P33.006	0.29	1	Pig	Feces
RM13_T4_B2	f__Spirochaetaceae (UID2512)	96.24	0.85	Treponema vincentii ATCC 35580	0.21	6	Human	Oral

RM4_T4_B5	f__Spirochaetaceae (UID2512)	98.66	1.21	Treponema vincentii ATCC 35580	0.23011	4	Human	the human oral cavity
RM7_T4_B4	f__Spirochaetaceae (UID2512)	98.66	1.21	Treponema vincentii ATCC 35580	0.23011	4	Human	Oral cavity
RM3_T6_B8	o__Clostridiales (UID1120)	84.49	0	uncultured Clostridium sp. strain UMGS1923	0.263022	2	Human	Gut
RM13_T6_B4	f__Porphyromonadaceae (UID2622)	97.01	0.47	uncultured Porphyromonas sp. strain UMGS547	0.17	15	Human	gut
RM4_T4_B6	f__Porphyromonadaceae (UID2622)	96.23	0.63	uncultured Porphyromonas sp. strain UMGS547	0.161822	17	Human	Gut
RM3_T6_B3	g__Prevotella (UID2721)	98.81	0	uncultured Prevotella sp. strain UMGS950	0.0690061	133	No listed	No listed
RM4_T6_B9	k__Bacteria (UID2329)	94.38	0.37	uncultured Sneathia sp. strain UMGS437	0.164662	16	Human	Vagina
RM12_T6_B5	p__Firmicutes (UID1022)	96.84	0	Veillonellaceae bacterium DNF00626	0.22	5	Human	Vagina
RM4_T4_B11	p__Firmicutes (UID1022)	94.26	0.57	Veillonellaceae bacterium DNF00626	0.219531	5	Human	vagina
RM4_T6_B5	p__Firmicutes (UID1022)	94.15	0.95	Veillonellaceae bacterium DNF00626	0.210897	6	Human	Vagina
