# Supplementary material for: Longitudinal Profiling of the Macaque Vaginal Microbiome Reveals Similarities to Diverse Human Vaginal Communities
Source: mSystems. 2021 Apr 27;6(2):e01322-20. doi: 10.1128/mSystems.01322-20 (PMC8092128; doi:10.1128/mSystems.01322-20)
Supplement: TABLE S1 [file mSystems.01322-20-st001.pdf]

Animal	Treatment group	Visit	Nugent Score	Clue Cells Present?	Gram Stain Comments	Vaginal pH	Whiff Positive?	Notes	*E2 (pg/ml)*	*P4 (ng/ml)*
RM1	Placebo	1.1	7	Y	coccis	6.5	Y		24	0.55
RM1	Placebo	2	4	N	coccis	7	N	spotting	63.68	<0.050
RM1	Placebo	3	4	N		7.5	N		35	<0.05
RM1	Placebo	4	6	Y	coccis, WBCs 3+	7.5	N		111.9	0.063
RM1	Placebo	5	8	Y	coccis	6.5	N		25.11	4.67
RM1	Placebo	6	9	Y	coccis, WBCs 2+	7	N		31.48	3.95
RM1	Placebo	7	4	N	coccis, mucus?	8	N		12.88	1.71
RM1	Placebo	8	8	Y	coccis, WBCs 3+	5.5	N		14.76	0.661
RM2	Placebo	1.1	10	Y	coccis	6	Y (SLIGHTLY)		50.91	9.66
RM2	Placebo	2	6	N	coccis	7	N		40.94	<0.050
RM2	Placebo	3	4	N		8	N		<5	<0.05
RM2	Placebo	4	6	Y	coccis, WBCs 4+	7	N		43.71	<0.050
RM2	Placebo	5	9	Y	coccis	8	N		10.15	3.48
RM2	Placebo	6	10	Y	coccis	5.5	N		48.73	5.2
RM2	Placebo	7	8	N	coccis, WBCs	7	N		18.63	1.49
RM2	Placebo	8	10	Y	coccis	7.5	N		282.9	0.124
RM3	Placebo	1.1	6	N	some diplococci	5.5	N		74	<0.05
RM3	Placebo	2	6	Y		5	N		29.43	0.139
RM3	Placebo	3	5	N		8	N		<5	<0.05
RM3	Placebo	4	4	N	coccis, WBCs 3+	8	N		46.06	<0.050
RM3	Placebo	5	3	N	WBCs 3+, diplococci	5	N		56.27	<0.050
RM3	Placebo	6	2	N	coccis, diplococcis, WBCs 2+	5	N		753.8	4.94
RM3	Placebo	7	0	N	coccis	5	N		12.69	1.26
RM3	Placebo	8	8	Y	coccis, WBCs 3+	6.5	Y		60.65	<0.050
RM4	Sucrose	1.1	10	Y	coccis	7.5	Y		33	<0.05
RM4	Sucrose	2	10	Y	coccis	7	N		82.03	<0.050
RM4	Sucrose	3	10	Y	coccis	8.5	N	light	<5	<0.05
RM4	Sucrose	4	10	Y	coccis	7.5	Y		43.8	<0.050
RM4	Sucrose	5	9	Y	ong thin curved neg rods like	7	N		54.62	<0.050
RM4	Sucrose	6	9	Y	coccis	8	N		91.7	<0.050
RM4	Sucrose	7	10	Y	coccis	7.5	N	spotting	37.15	6.17
RM4	Sucrose	8	10	Y	ong thin curved neg rods like	8	N		38.04	0.052
RM5	Sucrose	1.1	8	Y		7	Y		34	<0.05
RM5	Sucrose	2	5	N	coccis	8	N		73.42	3.91
RM5	Sucrose	3	4	N	coccis, WBCs? Mucus?	7.5	N	menstruating	68	<0.05
RM5	Sucrose	4	4	N	WBCs 4+	7	N		418.7	0.191
RM5	Sucrose	5	6	Y	WBCs 2+, coccis	5.5	N		76.12	10.43
RM5	Sucrose	6	8	Y	coccis	6	N		77.14	9.58
RM5	Sucrose	7	10	Y	coccis	7	N		58.78	<0.050
RM5	Sucrose	8	9	Y	coccis	5	N		79.16	7.42
RM6	Sucrose	1.1	10	Y	coccis	7	N		144	<0.05
RM6	Sucrose	2	5	N	coccis	9	N	spotting	72.5	<0.050
RM6	Sucrose	3	3	N	coccis	7.5	N	spotting	8	<0.05
RM6	Sucrose	4	4	N	coccis, WBCs 3+	7.5	N		84.45	<0.050
RM6	Sucrose	5	6	Y	coccis, WBCs 2+, mucus?	7	N		9.89	2.05
RM6	Sucrose	6	9	Y	coccis, WBCs 1+	6	N		36.82	7.32
RM6	Sucrose	7	9	Y	coccis	6.5	Y		72.28	3.95
RM6	Sucrose	8	9	Y	coccis, WBCs	7.5	Y		24.92	7.58
RM7	Sucrose	1.1	9	Y	coccis	7.5	Y		39	5.56
RM7	Sucrose	2	10	Y		6.5	Y		63.27	1.63
RM7	Sucrose	3	6	Y	coccis	7.5	N	menstruating	35	<0.05
RM7	Sucrose	4	9	Y	ong thin curved neg rods like	6.5	Y		47.13	<0.050
RM7	Sucrose	5	9	Y	coccis	7	Y		32.75	2.51
RM7	Sucrose	6	10	Y	coccis	7.5	Y		57.62	8.73
RM7	Sucrose	7	10	Y	coccis	7	Y		29.37	1.12
RM7	Sucrose	8	10	Y	coccis	7.5	Y		34.33	0.316
RM8	Placebo	1.1	8	Y	WBCs, coccis	6	N		28	4.25
RM8	Placebo	2	9	Y	coccis	5.5	Y		48.64	5.02
RM8	Placebo	3	10	Y		7	N	spotting	6	<0.05
RM8	Placebo	4	7	N	coccis, WBCs	7	Y	spotting	58.12	<0.050
RM8	Placebo	5	8	Y	WBCs 4+, coccis	6.5	Y		25.12	0.473
RM8	Placebo	6	7	Y	coccis, WBCs 3+	7	Y		42.23	6.52
RM8	Placebo	7	9	Y	coccis	7	Y		32.91	8.21
RM8	Placebo	8	10	Y	ong thin curved neg rods like	7	Y	spotting	178.5	<0.050
RM9	Placebo	1.1	4	N	RBCs?	7	N	mense	38	2.11

RM9	Placebo	2	7	Y	coccis	8.5	N		72.78	<0.050
RM9	Placebo	3	4	N	coccis	6.5	N		42	<0.05
RM9	Placebo	4	6	Y	coccis, WBCs	6.5	Y		43.53	<0.050
RM9	Placebo	5	7	Y	coccis	6.5	Y		530.9	0.635
RM9	Placebo	6	8	Y	coccis, WBCs 3+	8.5	N		47.64	4.02
RM9	Placebo	7	4	N	coccis	7.5	N	frank mense	36.44	6.12
RM9	Placebo	8	6	Y	coccis	5.5	Y		64.07	0.089
RM10	Sucrose	1.1	10	Y	coccis	6	Y	mense	43	0.55
RM10	Sucrose	2	8	Y	coccis	6.5	Y		49.83	<0.050
RM10	Sucrose	3	7	N	coccis, WBCs	7	N		23	<0.05
RM10	Sucrose	4	7	Y	coccis, WBCs 3+	7	Y		78.79	<0.050
RM10	Sucrose	5	10	Y	coccis	7.5	Y		22.22	3.36
RM10	Sucrose	6	10	Y	coccis	7	Y		27.37	4.64
RM10	Sucrose	7	9	Y	in curved neg rods like spagh	6.5	Y		25.91	0.509
RM10	Sucrose	8	9	Y	thin curved neg rods like spag	7	Y		12.07	3.54
RM11	Placebo	1.1	9	Y		6.5	Y		84	<0.05
RM11	Placebo	2	4	N	coccis	7	N	enstruating-fran	63.28	0.729
RM11	Placebo	3	9	Y	coccis	8	N		18	<0.05
RM11	Placebo	4	6	Y	coccis, WBCs 2+	7	Y		92.75	<0.050
RM11	Placebo	5	10	Y	coccis	7	N		14.71	1.85
RM11	Placebo	6	10	Y	coccis 4+	7	Y		21.82	4.45
RM11	Placebo	7	10	Y	coccis, WBCs	7.5	Y		41.15	2.55
RM11	Placebo	8	9	Y	coccis, WBCs	7	N		133.7	0.086
RM12	Sucrose	1.1	9	Y	coccis	7	Y		26	0.58
RM12	Sucrose	2	9	Y	coccis	7	N		38.11	4.37
RM12	Sucrose	3	4	N		8.5	N	light	<5	<0.05
RM12	Sucrose	4	6	N	coccis, WBCs 3+	6.5	N		33.11	<0.050
RM12	Sucrose	5	7	Y	s, long thin curved neg rods l	7	N		336.9	0.315
RM12	Sucrose	6	8	Y	coccis	5	N		24.92	3.32
RM12	Sucrose	7	5	N	coccis, WBCs 3+	7	N		33.28	4.85
RM12	Sucrose	8	10	Y	ng thin curved neg rods like s	7.5	N		49.38	0.074
RM13	Placebo	1.1	9	Y	coccis	7	Y (VERY)	ells sour/like pick	27	<0.05
RM13	Placebo	2	10	Y	coccis	7.5	N		36.1	<0.050
RM13	Placebo	3	9	Y	coccis 4+, WBCs	7	Y	spotting	<5	<0.05
RM13	Placebo	4	9	Y	thin curved neg rods like spag	6.5	Y		41.67	<0.050
RM13	Placebo	5	10	Y	coccis	7.5	Y		<5	0.568
RM13	Placebo	6	9	Y	coccis, WBCs 2+	7	Y		37.84	4.13
RM13	Placebo	7	9	Y	coccis	6.5	Y		45.02	5.79
RM13	Placebo	8	9	Y	ng thin curved neg rods like s	7.5	Y		51.49	<0.050
RM14	Sucrose	1.1	7	Y		6.5	Y	spotting	41	4.89
RM14	Sucrose	2	7	Y		7	N	enstruating-ligh	31.24	6.01
RM14	Sucrose	3	4	N		8	N	spotting	30	<0.05
RM14	Sucrose	4	4	N	WBCs, coccis in chains	5.5	N	us with some bl	85.9	0.074
RM14	Sucrose	5	4	N	coccis	4.5	N		55.45	2.86
RM14	Sucrose	6	4	N	coccis, WBCs 1+	4.5	N		18.5	6.64
RM14	Sucrose	7	4	N	coccis	7	N	spotting a little	64.49	0.161
RM14	Sucrose	8	9	Y	coccis	4.5	N		19.19	5.58
RM15	Sucrose	1.1	4	N	diplococci	8	N		43	<0.05
RM15	Sucrose	2	10	Y	coccis	6.5	Y		26.27	0.629
RM15	Sucrose	3	4	N	coccis	8	N	spotting	36	<0.05
RM15	Sucrose	4	4	N	WBCs 3+	6	N		61.04	0.063
RM15	Sucrose	5	5	N	coccis, WBCs	5.5	N		27.43	4.31
RM15	Sucrose	6	8	Y	coccis	6.5	Y		15.21	3.99
RM15	Sucrose	7	5	N	coccis	6.5	N	spotting	8.54	0.063
RM15	Sucrose	8	4	N	coccis, WBCs 4+	4.5	N		43.58	<0.050
RM16	Placebo	1.1	10	Y		7	N		63	0.06
RM16	Placebo	2	9	Y		6.5	Y		76.14	6.39
RM16	Placebo	3	6	N	lococcis; odd slide, not a lot	8.5	N	spotting	66	<0.05
RM16	Placebo	4	4	N	WBCs	6.5	N		89.07	<0.050
RM16	Placebo	5	7	Y	coccis, WBCs 2+	6.5	N		21.93	1.14
RM16	Placebo	6	9	Y	coccis, WBCs 2+	6.5	N		30.37	7.03
RM16	Placebo	7	5	N	coccis	7	N	spotting	59.22	11.98
RM16	Placebo	8	9	Y	coccis, WBCs	6	N		332.3	0.075
